# Exploring the Effects of Argon Plasma Treatment on Plasmon Frequency and the Chemiresistive Properties of Polymer-Carbon Nanotube Metacomposite

**DOI:** 10.3390/ma10090986

**Published:** 2017-08-24

**Authors:** Manuel Rivera, Mostafizur Rahaman, Ali Aldalbahi, Rafael Velázquez, Andrew F. Zhou, Peter X. Feng

**Affiliations:** 1 Department of Physics, University of Puerto Rico, San Juan, PR 00936-8377, USA; manuel.rivera3@upr.edu (M.R.); r.velazquez.vicente@gmail.com (R.V.); 2 Department of Chemistry, College of Science, King Saud University, Riyadh 11451, Saudi Arabia; mrahaman@ksu.edu.sa; 3 Department of Physics, Indiana University of Pennsylvania, 975 Oakland Ave, Indiana, PA 15705, USA; fzhou@iup.cdu

**Keywords:** metacomposite, polymer, carbon nanotube, gas sensor, plasmon frequency, dielectric spectroscopy

## Abstract

Metacomposites, composite materials exhibiting negative permittivity, represent an opportunity to create materials with depressed plasmon frequency without the need to create complex structural geometries. Although many reports exist on the synthesis and characterizations of metacomposites, very few have ventured into exploring possible applications that could take advantage of the unique electrical properties of these materials. In this article, we report on the chemiresistive properties of a polymer-CNT metacomposite and explore how these are affected by Argon plasma treatment.

## 1. Introduction

In recent years, several reports have surfaced regarding various nanocomposite materials exhibiting a negative permittivity of significantly high magnitude [1,2]. As a result, the term metacomposites has been introduced to describe these unique nanocomposites with negative permittivity, which are promising candidates for applications such as super lenses, wave filters, remote aerospace applications, and superconductors. Among these reports, it was observed that the negative permittivity and the plasmon frequency observed in conductive polymer nanocomposites (PNCs) can be tuned by changing the filler loading levels, the ratio of oxidant to the monomer and nanofiller morphologies. Since the mid-90s [3], the search for negative index materials or metamaterials with plasmon frequencies depressed to at least the far infrared or even the GHz range has been pursued mostly by designing materials with complicated geometrical structures [4], and PNC metacomposites offer a chemical formulation route for creating negative index materials which could potentially simplify and lower the costs of fabrication [4,5]. Additionally, compared to negative index materials based on complex architectures (i.e., metamaterials), metacomposites exhibit a certain robustness and tolerance to an array of chemical and physical treatments, and as a result, they are more capable of withstanding modifying procedures such as chemical functionalization or plasma surface treatment undertaken in an attempt to tune desirable properties without compromising the integrity of the material. 

It is reasonable to expect unique chemiresistive properties among some of these metacomposites. However, even though both polyaniline (PANI) and carbon nanotubes (CNTs) have been explored as potential sensing materials in gas sensors [6,7], to the best of our knowledge there have been no attempts to investigate their chemiresistive properties or feasibility as gas sensing materials once they have been successfully combined into a PNC metacomposite. In the present paper, we report on the chemiresistive properties of PANI/multi-walled carbon nanotube (MWCNT) nanocomposite doped with anionic biopolymer iota-carrageenan (IC) exhibiting a negative permittivity. Argon non-thermal plasma surface treatment was performed on the IC/PANI/MWCNT (ICPM) metacomposite, and its effects were analyzed by Raman spectroscopy. Plasma treatment has been known to change the chemiresistive properties of CNTs and polymers [7,8]. Characterization of the electrical properties of the ICPM was performed before and after plasma treatment, and its effects on the chemiresistive properties and the plasmon frequency were investigated.

## 2. Experimental Details

The ICPM metacomposite was synthesized by chemical polymerization of aniline in the presence of MWCNTs. 3 mg of MWCNTs were dispersed in 2 mL of IC solution (0.5% *w*/*v*) by sonicating for 40 min using a digital sonicator (Branson450, 400 W, Fisher Scientific, Asheville, NC, USA). 0.2 mL of aniline was added and the mixture was stirred on a hot plate stirrer at 21 °C for 2 h. 2 mL of IC (0.5% *w*/*v*) solution was added to the mixture which was subsequently stirred for 1 h followed by sonication in a bath sonicator (Unisonics, Brookvale, NSW, Australia) in an ice water bath for 6 h. 0.49 g of ammonium persulfate was dissolved in 1 mL of Milli-Q water and was added dropwise to the above mixture under stirring on a hot plate stirrer for 24 h at 21 °C. The composites were filtered, using a nylon membrane and washing several times with Milli-Q water and methanol. The product was dried in a vacuum oven at 60 °C for 24 h. Electrical current and voltage (I-V) curves were obtained by monitoring the resistance using a Keithley multimeter (Tektronix Inc., Beaverton, OR, USA) and dielectric measurements by using a Novocontrol Broadband Dielectric Spectrometer (Novocontrol Technologies, Montabaur, Germany). The ICPM metacomposite was treated with 30 short pulses (pulse width: ~10 microseconds, frequency: 1 Hz**)** of Argon gas plasma beams with a discharge voltage of 500 V.

Gas sensing measurements were performed in a sealed cryostat under different conditions for comparison. Firstly, the chamber containing the sample and electronic measurements setup was filled with room temperature air at atmospheric pressure and percent relative humidity (RH%) of around 55%. I-V curves were obtained immediately after these conditions were met. After this, a vacuum at 5.0 × 10^−3^ torrs of pressure was created inside the chamber and sealed from the environment. Once this was achieved, we proceeded to measure the I-V curves. Similarly, the chamber under vacuum was filled with hydrogen (purity ≥ 99.996%) at 760 torrs. Electric measurements were only recorded after the hydrogen-filled chamber reached the specified pressure. Hence, measurements were performed under conditions of equilibrium, and not under constant gas flow.

## 3. Findings

**Figure 1:**
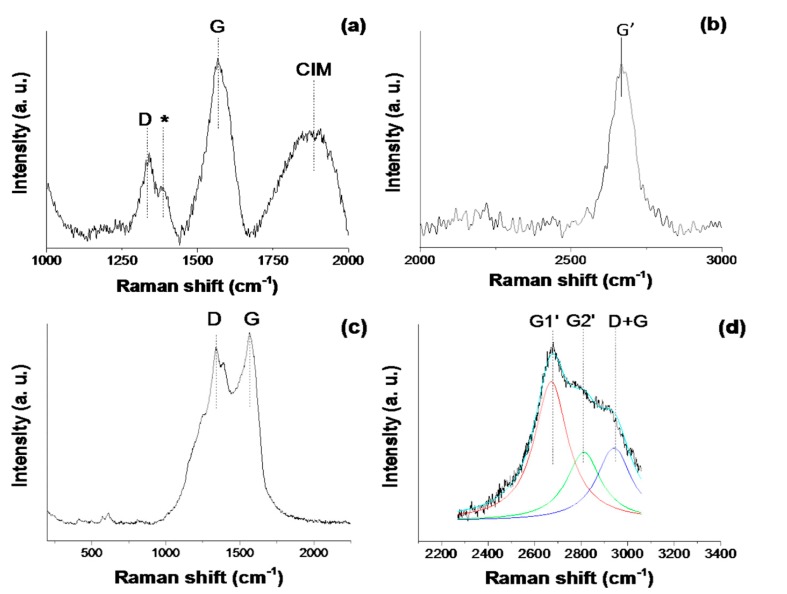
Raman Spectra of IC/PANI/MWCNT (ICPM) metacomposite before (**a**,**b**) and after (**c**,**d**) plasma treatment.

Figure 1a,b represent different ranges of the Raman spectrum of the ICPM metacomposite before plasma surface treatment. Typical signature peaks of carbon nanotubes, the D and G bands, are clearly visible, and are positioned at 1300 cm^−1^ and 1580 cm^−1^, respectively [9]. The peak that appears around 1400 cm^−1^ (marked with an asterisk), and which merges with the D band, is attributed to PANI and its interaction with CNT [10].

The Raman spectrum of the untreated sample exhibits an additional intense and broad peak centered at 1800 cm^−1^. The presence of this peak, known as the coalescence–inducing mode (CIM), in the Raman spectrum of MWCNTs has been considered to be due to linear carbon chains with pure sp hybridization known as carbyne molecules [11,12]. The existence of such carbyne molecules has been debated in the past due to the fact that they would be highly unstable [11]. However, CNTs might provide a protective environment in which these forms of carbon are able to persist. The second-order peak at ~2700 cm^−1^ (Figure 1b), typically called the G’ band, corresponds to the overtone of the D band [13].

The Raman spectrum of the ICPM composite treated with pulsed argon plasma beams is shown in Figure 1c,d. It is clear from comparing Figure 1c with Figure 1a that the I_D_/I_G_ ratio has increased due to the plasma treatment. An increase in the I_D_/I_G_ ratio is consistently observed upon functionalization of CNTs [14]. Stronger interactions between the CNTs, PANI and IC induced by the plasma treatment might have contributed to the broadening and the apparent merging of the D and G modes [10]. Given that carbyne molecules are highly reactive, unstable, and are easily transformed into other common carbon structures, the fact that the CIM mode is no longer observed in the Raman spectrum suggests that the linear carbon chains were “destroyed” upon exposure to the plasma energy [15].

The second order features of the Raman spectrum, Figure 1d, have also changed due to the plasma treatment. We observe a splitting of the G’ band into two peaks, G1’ and G2’, positioned at 2671 cm^−1^ and 2811 cm^−1^, respectively, plus the nearby D + G mode situated at around 2900 cm^−1^. The two-peak structure of the G’ mode in graphitic structures has been attributed to interlayer coupling and stacking order of 3D graphitic material [9,13] present in a material and/or Fermi-level downshifts due to doping procedures performed on CNTs [16,17]. A downshift in Fermi level has been shown to increase conductivity and Drude plasmon frequency in treated CNTs [17]. The D + G band observed in Figure 1d is typically a distinguishable mode between pristine and functionalized MWCNTs [14,18]. In Figure 1c, we can also observe two additional small peaks at the low-energy end of the spectrum, positioned at 575 cm^−1^ and 615 cm^−1^, respectively. These are signature peaks of I-carrageenan, and are assigned to two different υ_4_ vibrations of the sulphate group, primarily O=S=O bending vibrations. [19] 

**Figure 2:**
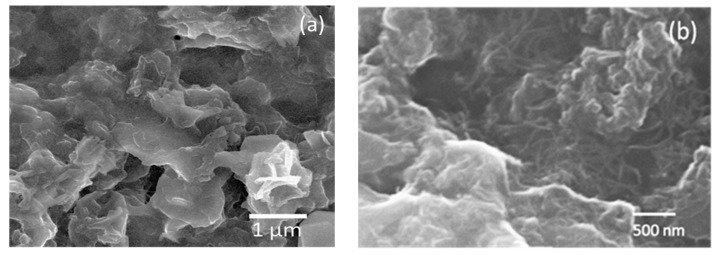
SEM image of (**a**) untreated ICPM and (**b**) argon plasma-treated ICPM metacomposite.

The SEM image in Figure 2b of the treated ICPM reveals a rough porous surface, observed as cavern-like structures. This has the desirable effect of increasing the overall interphase area between the sample and any gas it could potentially detect. Also, MWCNTs can be seen in Figure 2b as numerous fiber-like structures which create an apparent network covering most of the surface area. This is indicative that a good dispersion of MWCNTs was achieved. Carrageenan biopolymer has been observed in the past to facilitate MWCNT dispersion [20]. Similar features were observed in the untreated sample shown in Figure 2a.

**Figure 3:**
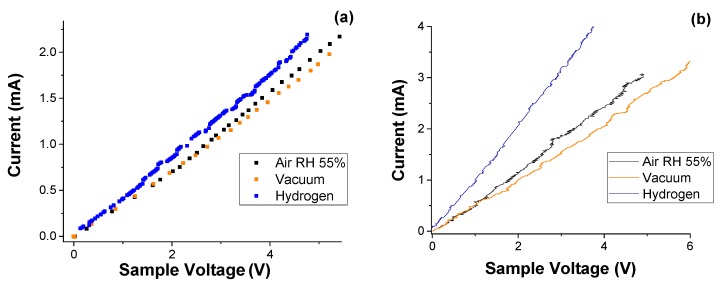
Electrical current as function of the applied voltage of (**a**) untreated and (**b**) treated ICPM composite at room temperature.

Figure 3 shows the I-V characteristics at room temperature (24 °C) of the ICPM metacomposite before and after the argon plasma treatment. Measurements were taken separately in ambient air with approximately 55% RH, in the vacuum, and in the presence of H_2_ gas at a pressure of 1 atm.

We can see in Figure 3 that the ICPM metacomposite has an ohmic electrical behavior under all measured conditions. From Figure 3a it is clear that the material is unsuitable for hydrogen gas or humidity sensing. The current-voltage response of the plasma-treated ICPM is shown in Figure 3b. Compared to the untreated ICPM metacomposite, Argon plasma treatment resulted in an overall increase in conductivity of the sample; 36% increase in the case of ambient air and 28% increase when placed under vacuum. Sensitivity to humidity, compared to vacuum, does not appear to increase significantly upon plasma treatment. This observation is important, due to the fact that many gas sensing materials experience sensitivity degradation due to the presence of humidity. In contrast, the post-plasma treatment sample (Figure 3b) responded significantly to H_2_ gas over both vacuum and ambient air. Applying the typical definition of sensitivity,

(1) $$S=\frac{\left|R_{a}-R_{h}\right|}{R_{a}}\times 100,$$

where *R_a_* is the resistance in humid air and *R_h_* is the resistance in the hydrogen atmosphere, a saturated sensitivity to hydrogen gas of approximately 41% (higher in dryer environments) was obtained from the plasma-treated ICPM data presented in Figure 3b, as opposed to less than 8% exhibited in the untreated sample.

Since the mid-90s, studies on the effects of plasma treatment on polymers have been carried out, and have opened up an effective and simple route to modify and alter many chemical and physical properties of polymers [21,22,23]. It has been found that activated species present in the plasma discharge, such as electrons, ions and ultra violet (UV) photons, will break C–C or C–H bonds, leading to the production of carbon radicals [24]. These carbon radicals are the initiators of intra- and interchain chemical step reactions, resulting in a cross-linked network on the polymer surface and surface oxidation through the incorporation of oxygen functionalities from residual oxygen present in the chamber [25,26]. In most cases, the macro effect appears to be an increase in hydrophilicity and adhesion improvement of the plasma-treated polymer [8,22,23]. On the other hand, depending on the plasma gas mixture applied, the incorporation of different functionalities also appears in CNTs upon plasma treatment [24,27]. The increase in the Id/Ig ratio in combination with the appearance of the D + G second order mode and the apparent merging of the D and G modes in the Raman spectrum (Figure 1c) of the plasma-treated sample are indicators of the presence of a variety of functional groups and enhanced MWCNTs-polymer matrix interactions [10,28]. Crosslinking within polymer chains and with MWCNTs caused by the incorporation of plasma-induced functionalities could possibly open up additional conduction paths, increasing the conductivity of the material as a result. Additionally, it is known that the noise level in an electrical signal from a MWCNT-polymer composite increases with the number of nanotube-polymer interphases present in the conduction paths [7]. The increase in noise as a result of plasma treatment, seen in Figure 2b, also suggests that MWCNTs-polymer interactions have been enhanced, and as a result, there is a larger number of interphases present in the conduction paths. Therefore, the exact sensing mechanism is extremely difficult to determine, given that it might have contributions from multiple processes. More research is required to address this issue.

Dielectric spectroscopy (DS) measurements in the frequency range of 10^−1^–10^6^ Hz were performed on the ICPM metacomposite before and after plasma treatment.

**Figure 4:**
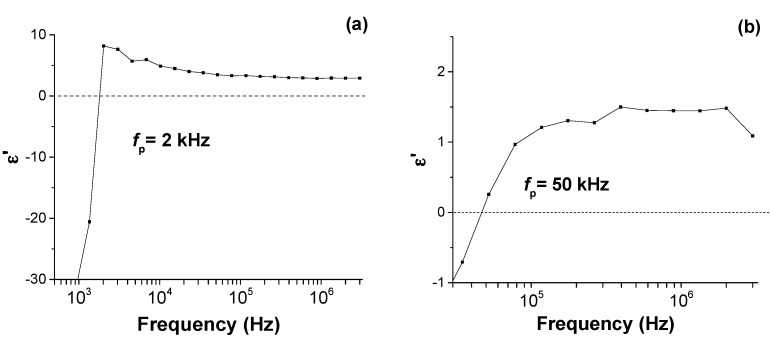
Real permittivity for (**a**) nontreated or pristine ICPM metacomposite and (**b**) plasma-treated IPM in the frequency range close to *f_p_*.

Figure 4 shows the results of the DS measurements in the frequency range close to the plasmon frequency. Negative permittivity in metacomposites is considered to be the result of network formation of continuous conducting pathways that are capable of generating delocalized charges on a macroscopic scale [5]. The negative permittivity in such systems is commonly described by the Drude Model according to the following formula: [1,4]

(2) $$\varepsilon^{\prime }\left(f\right)=1-\frac{f_{p}^{2}}{f^{2}+\gamma^{2}}$$

where *f* is the frequency of the applied electric field, *f_p_* is plasmon frequency and *γ* is the dissipation parameter. Additionally, the model derives a relationship between *f_p_* and the charge carrier density *N*:

(3) $$f_{p}=\frac{2Ne^{2}}{m_{eff}}$$

where *m_eff_* is the effective mass of the electron and *e* the electron charge. Since the dissipation parameter *γ*^2^ is considered negligible, from Equation (2) it can be seen that ε’ (*f_p_*) = 0. Analysis of the data shown in Figure 4a reveals that the untreated sample had *f_p_* = 2 kHz, while *f_p_* in the treated sample, Figure 4b, has shifted to 50 kHz. According to equation 3, plasma surface treatment has resulted in increasing the charge carrier density *N* [29]. 

Reports on nanocomposites exhibiting negative permittivity have found that plasmon frequency can be tuned by functionalizing one or more of its components and/or increasing the filler concentration [29,30,31]. For instance, Zhu et al. [32] investigated the effects of carbon filler structure and concentration in PANI nanocomposites on the dielectric properties. They found by using different carbon fillers, such as CNT, graphene and carbon nano fibers, that carbon structures with higher filler-polymer interactions resulted in higher plasmon frequency *f_p_* of the PANI nanocomposite. Increase in filler content, independently of the filler used, increased the plasmon frequency as well. The emergence of negative permittivity in polymer-nanocarbon composites as carbon filler concentration increases, as well as the increase in the Drude plasmon frequency, is typically attributed to the percolation phenomenon [30], where a critical concentration value—the percolation threshold—needs to be attained in order for a conduction network to be formed. The plasmon frequency of the IPM composite in this work increased by a factor of 25 due to the incorporation of functionalities and improved cross-linkage between the CNTs and the polymer matrix by argon plasma treatment, which is a significantly higher increase than that obtained in any other study reporting changes in *f_p_* of PNC metacomposites. Moreover, plasma treatment is a much more cost-effective and non-hazardous means to increase plasmon frequency in metacomposites than increasing filler concentration or chemically treating the components in order to introduce functional groups. 

Plasma treatment appears to have the additional effect of tuning the chemiresistive properties of the IPM composite towards increased H_2_ sensitivity. A negligible sensitivity to hydrogen was observed in the untreated sample. Upon plasma treatment (Figure 3b), the saturated sensitivity to hydrogen increased by about 41%. This result reinforces the previously published observation that plasma treatment is an advantageous effective procedure for amplifying the gas sensitivity of materials through the incorporation of functional groups [8,33]. However, in contrast to these reports, the increased sensitivity of hydrogen observed in the treated sample was highly selective, as no significant changes were observed related to the material’s sensitivity to humidity. This suggests that plasma treatment is potentially capable of amplifying the desired properties of gas sensing materials without affecting others, hence opening up the attractive possibility of finding suitable metacomposites to be used as gas sensing material with sensing properties related to the value of the plasmon frequency.

## 4. Conclusions

An ICPM metacomposite exhibiting negative permittivity was successfully synthesized, characterized and current-voltage tested before and after argon plasma surface treatment. The objective was to explore, for the first time, the chemiresistive properties of a metacomposite and their relationship to its plasmon frequency. The observed negative permittivity was described according to the Drude model. We observed a significant increase in conductivity and sensitivity to hydrogen gas as a result of the plasma treatment while remaining “blind” to humidity. Additionally, plasma treatment had the effect of increasing the Drude plasmon frequency from 2 to 50 kHz. To the best of our knowledge, this is the highest shift in plasmon frequency reported due to any type of modification or treatment performed on a metacomposite. This suggests that the chemiresistive properties determining the sensitivity and selectivity of a potential gas sensing metacomposite could possibly be tuned by manipulation of the plasmon frequency. 

Needless to say, if any chemirestive property of a metacomposite, or changes in chemiresistive properties due to some physical or chemical treatment, could effectively be predicted or represented by the value and/or shifts in value of the plasmon frequency, would be of paramount importance. Therefore, more research on the subject is highly needed and desirable. Additionally, further exploration of new possible applications of metacomposites that exploit their unique electrical properties as well as developing and applying modification techniques such as plasma treatment as tinkering tools are much needed.
